# Health Care Professionals’ Experiences and Views of eHealth in Pediatric Care: Qualitative Interview Study Applying a Theoretical Framework for Implementation

**DOI:** 10.2196/47663

**Published:** 2023-10-18

**Authors:** Charlotte Castor, Rose-Marie Lindkvist, Inger Kristensson Hallström, Robert Holmberg

**Affiliations:** 1 Department of Health Sciences Lund University Lund Sweden; 2 Department of Psychology Lund University Lund Sweden

**Keywords:** communication, digital, experiences, eHealth, health care professionals, implementation, NASSS, pediatric care

## Abstract

**Background:**

The development and evaluation of eHealth interventions in clinical care should be accompanied by a thorough assessment of their implementation. The NASSS (Non-adoption, Abandonment, and Challenges to the Scale-Up, Spread, and Sustainability of Health and Care Technologies) framework was designed to facilitate the implementation and scale-up of health technology programs, providing an option for analyzing the progression of these initiatives as they are implemented in real-time. Considering health care provider perspectives within the framework for implementation offers valuable insights into the early identification of barriers and facilitators in the implementation of potentially effective eHealth innovations. Nevertheless, there is a dearth of studies on eHealth interventions that encompass longer time frames and delve into the complexities of scaling up and sustaining such interventions within real-world health care environments.

**Objective:**

This study aims to investigate the perspectives and insights of health care professionals (HCPs) regarding the implementation of an eHealth intervention in pediatric health care while applying the NASSS framework to theorize and evaluate the conditions influencing the implementation of eHealth solutions.

**Methods:**

Semistructured interviews were performed with health care providers, including both staff and management personnel, within a university pediatric hospital (N=10). The data collection process occurred concurrently with a clinical trial focused on developing and assessing an eHealth app for self-management in pediatric care following hospital discharge. Using an abductive approach, the interviews were initially analyzed qualitatively and subsequently mapped onto the 7 domains of the NASSS framework to identify factors influencing implementation, encompassing facilitators, barriers, and varying levels of complexity.

**Results:**

In the realm of pediatric care, the family was identified as the primary unit of care, and patient heterogeneity was a prominent feature. The implementation of eHealth tools, while deemed usable and flexible, was also seen as a delicate balance between safety and adaptability, highlighting challenges related to health care integration. Child participation and secrecy, especially for adolescents, contributed to the complexity of using eHealth. HCPs had high eHealth literacy, and thus challenges concerning adoption were related to work adaptations and the risk of “app overload.” The readiness for implementation was experienced as induced through the research study and the pandemic situation. However, to move from research to implementation in clinical practice, organizational challenges identified a need to update the concept of care and ensure activity measurements. In a wider context, HCPs raised concerns related to regulatory requirements for documentation, public procurement, and data safety. Implementation became more complex due to a lack of overview in a large organization.

**Conclusions:**

Important perspectives for implementation were considerations of regulatory requirements, as well as the need for a shared vision of eHealth and the establishment of eHealth-related work as part of regular health care. Key contextual factors that support reach and impact are communication channels between different levels at the hospital and a need for paths and procedures compatible with legal, technological, and security concerns. Further research should focus on how eHealth interventions are perceived by children, adolescents, their parents, and other stakeholders.

**Trial Registration:**

ClinicalTrials.gov NCT04150120; https://clinicaltrials.gov/ct2/show/NCT04150120

## Introduction

Technological development and increased access to the internet and mobile technology have led to a flurry of eHealth interventions to support families and children in pediatric care [1-3]. The general purpose of eHealth, defined as the use of information and communication technologies for health [4], is to facilitate high-quality and equal care and health for populations through, for example, increased access to care and improved health information exchange. Many countries have adopted national eHealth policies or strategies aiming to enhance person-centered care [5].

To ensure that the goals of eHealth are met, interventions that are developed and scientifically evaluated need to be studied from the perspective of a real-world context. Recent studies have raised the importance of attending to the complexity of the context of these interventions to support a move from innovation to implementation [6,7].

The NASSS (Non-adoption, Abandonment, and Challenges to the Scale-Up, Spread, and Sustainability of Health and Care Technologies) framework is a validated tool developed to support the implementation and scale-up of health technology programs, offering a structure for studying the unfolding of such initiatives in 7 domains (Textbox 1) [8].

**The 7 domains in the NASSS (Non-adoption, Abandonment, and Challenges to the Scale-Up, Spread, and Sustainability of Health and Care Technologies) framework.**

1. The condition or illness
2. The technology
3. The value proposition
4. The adopter system
5. The organization
6. The wider context
7. Embedding and adaptation over time

The NASSS framework has been used in numerous studies, incorporating qualitative data such as interviews and focus group discussions. These studies have used a combination of inductive and deductive approaches to inform their analyses [9-12]. Thus, the framework can serve as a guiding tool for development and decision-making. It can assist in avoiding common high-probability issues and, in certain situations, prompt discussions about discontinuing high-risk projects rather than continuing to invest resources.

The insights provided by health care professionals (HCPs) in various roles within the health care system are crucial for the advancement and integration of innovative care delivery methods. The NASSS framework has proven valuable in structuring and contrasting HCPs’ encounters with eHealth, thus revealing potential intricacies and obstacles in the adoption and dissemination of such technologies. Examining the broader landscape beyond individual projects can offer valuable perspectives for strategic planning and implementation, with a particular focus on understanding how contextual factors can introduce complexities that either facilitate or impede the adoption and expansion of innovative solutions [13].

Interventions incorporating eHealth technologies are inherently multifaceted, entailing complexities associated with technology development, support, maintenance, and financing. Much of the existing research on eHealth interventions has concentrated on individual technologies and the challenges and enablers tied to their implementation [8]. A systematic review of the literature highlighted substantial gaps and obstacles concerning the implementation of eHealth and called for a more comprehensive analysis of the experiences of various stakeholders [14]. Relatively few studies have delved into longer time frames that encompass the scaling up and long-term sustainability processes within the intricate landscape of real-world health care environments [8].

The objective of this study was to investigate the experiences and perspectives of HCPs regarding the implementation of an eHealth intervention in pediatric health care, using the NASSS framework as a theoretical lens to conceptualize and assess the factors influencing eHealth implementation

## Methods

### Design

We used a qualitative research design with a descriptive abductive approach rooted in naturalistic inquiry [15]. Qualitative data gathered through focus groups and individual interviews with HCPs were initially analyzed using an inductive approach. Subsequently, these findings were applied deductively within the NASSS framework to elucidate the factors influencing the implementation of eHealth [8]. This study was conducted in parallel with a controlled experimental clinical trial [16], registered under ClinicalTrials.gov identifier NCT04150120. The trial design adhered to the Medical Research Council’s framework for complex intervention trials [6,17].

### Study Setting

The study was conducted from January to June 2021 in 4 pediatric departments located at a university hospital in southern Sweden. These departments provide care for children with severe illnesses at the local, regional, and national levels. The primary focus of the research revolved around an eHealth intervention developed as part of the eChildHealth research (eCH) program. The eCH program aimed to facilitate self-management for pediatric patients following their discharge from the hospital. It encompassed 4 specialties: pediatric surgery, neonatology, oncology, and cardiology [16]. The eCH program involved the development and evaluation of an app that was installed on tablets provided to families upon hospital discharge. Additionally, it included a web-based interface designed for HCPs to facilitate bilateral communication. The software offered a range of functionalities, such as daily reporting, video calls, and text messaging, among others.

### Participants

The study included a total of 10 participants, who were HCPs divided into 2 subsamples: (1) staff members from various professions directly engaged in the further development [16] and evaluation [18] of the eCH intervention; and (2) management personnel within the relevant pediatric departments. In the autumn of 2020, invitation letters for participation in focus group interviews were distributed via email to all staff members who had previously been involved in the development and evaluation phases of the eCH intervention. Those who did not initially respond received a follow-up email between 4 and 8 weeks later. In total, 15 pediatric nurses and physicians were invited to participate; 10 of them responded affirmatively to the initial invitation, and 5 were available for participation. In a subsequent step, all managers at different hierarchical levels within the pediatric departments (n=25) were contacted by email and invited to join a focus group interview. A reminder email was sent to those who did not respond, resulting in the participation of 5 managers.

### Data Collection

Because of the COVID-19 pandemic, data collection was planned and performed for digital interaction via Zoom (Zoom Video Communications), allowing for increased flexibility regarding scheduling and the number of participants. In total, the data collection involved 2 focus group interviews and 1 individual interview with 5 HCPs, as well as 5 individual interviews with managers. During the interviews, 2 out of 3 researchers responsible for data collection (CC, RH, and RML) were present, with each taking turns as the interviewer and notetaker. Structured interview guides were used, with separate versions for staff and managers. These guides included questions about participants’ general experiences with eHealth and their specific involvement in an ongoing clinical trial [16]. The interviews with staff began with an oral introduction explaining the study’s purpose, while the interviews with management included a brief presentation, supported by an MS PowerPoint slide show (Microsoft Corporation), introducing the concept of eHealth and the ongoing clinical trial [16]. The primary areas explored during the interviews were as follows: (1) What were the experiences and primary concerns of staff and management regarding the feasibility of implementing eHealth in routine care? (2) In which areas did their accounts of their involvement indicate a sense of knowledge and efficacy, and in which areas were they more uncertain or less prepared? Although the interviews initially focused on a specific clinical trial [16,18], they were conducted within a broader context of eHealth. The interviews had varying durations, ranging from 42 to 59 minutes.

### Analysis

Audio-recorded and transcribed interviews were analyzed in 3 separate analytical steps: 2 data driven and 1 theory driven. First, an inductive analysis of data was performed. Three of the authors (CC, RML, and RH) read the text of each interview and divided it into meaningful units, which were condensed and coded by CC and RML, following the initial steps of qualitative content analysis [19]. Second, codes were applied deductively using the NASSS framework [8] as a theoretical lens of analysis by identifying and organizing codes into the 7 domains of the NASSS by the 3 authors. All codes were considered and the NASSS framework was used as a guide to identify relevant codes. Codes not fitting into any of the domains of the NASSS, comprising background information about the participants and reflections related to the term eHealth or the scope of the clinical trial, were deemed impertinent to the analysis and therefore excluded. Third, codes within each domain were grouped based on content, and then summarized and presented with representative quotes. Parallel to this step, the level of complexity within each domain was considered based on the codes grouped into each separate domain. Finally, the result was discussed and agreed upon by all authors. Data were managed using the software Open Code (Umeå University) [20].

### Ethical Considerations

The study was approved by the Swedish Ethical Review Authority (approval number 2019-0341). All participants were given written information about the study in advance and additional oral information with the possibility of asking questions at the time of the interview. Voluntary participation was stressed, and informed consent was obtained. All participants were well acquainted with the research and its underlying principles. The authors possess extensive expertise in qualitative methods, implementation research, and nursing. Additionally, they were actively engaged in the research project that designed and assessed eCH, although none of the authors were directly involved in the application of eCH.

## Results

### Overview

**Figure 1:**
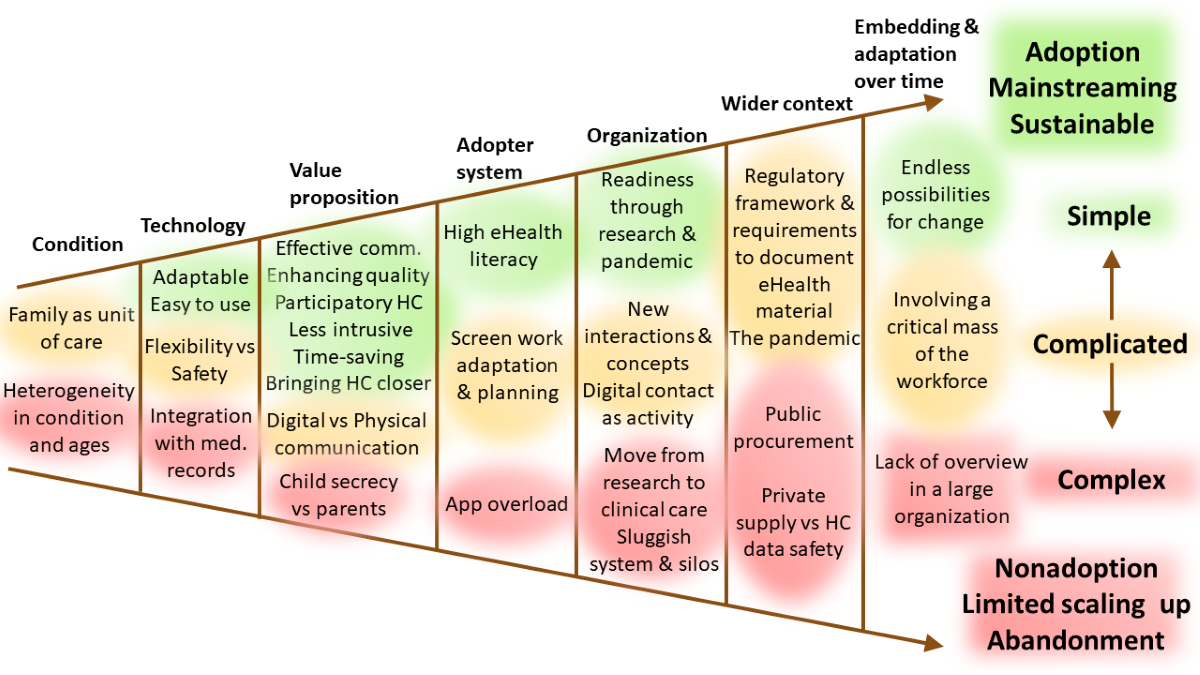
Health care professionals’ experiences and views on the eChildHealth intervention applied to NASSS conditions for implementation. comm: communication; HC: health care; med: medical; NASSS: Non-adoption, Abandonment, and Challenges to the Scale-Up, Spread, and Sustainability of Health and Care Technologies.

The presentation of HCPs’ perspectives on eHealth, structured based on the NASSS framework, encompassed both eHealth in a broader sense and various specific eHealth interventions, including eCH. Feedback related to eCH predominantly emanated from staff representing various professions directly engaged in the research, while managers shared more generalized experiences. Figure 1 visually depicts the continuum of simplicity (green), through complexity (yellow), to high complexity (red) in terms of implementation within each domain.

### Domain 1: The Condition or Illness

In their discussions about eHealth in general and specifically the eCH, HCPs highlighted a significant diversity of circumstances, encompassing care for neonates through to adolescents, with an emphasis on the family as the central unit of care. The presence of long-term and recurrent hospital admissions, in conjunction with outpatient care, added layers of complexity to the implementation of eHealth across the hospital setting. The HCPs recognized that children grappling with chronic conditions, such as cystic fibrosis, diabetes, cardiac diseases, anorectal malformation, or cancer, faced considerable challenges in their daily lives due to their illnesses. Consequently, there was a perceived necessity for eHealth initiatives that could facilitate communication and provide support to these children and their families in a home-based context.

What is so exciting about this project [eCH]...is that it’s specifically oriented towards children. We do not have any systems directed at children. We have to tweak many systems a little to make them fit children’s needs. This is developed for children.

Interview 3 staff

Furthermore, the HCPs noted that the diversity in family structures, language proficiency, health literacy, and socioeconomic status added to the intricacy of implementing eHealth interventions. They emphasized the importance of recognizing and addressing these variations when designing and delivering pediatric care.

### Domain 2: The Technology

The HCPs described eCH as accessible and ready to use with functions that could be activated or deactivated according to one’s needs. By contrast, one HCP described that too many functions could lead to confusion and the possibility of missing data.

When the system offers a great number of functions and the patient only uses some of them, then you might start to wonder. It´s a problem with ‘missing data’ really. Does the patient not feel any pain or is this [the technology] something the patient doesn’t need?

Interview 3 staff

The HCPs found that technical skills among staff were most effectively developed through hands-on experience and with support from reliable supplier companies. They highlighted the importance of eCH being developed and adjusted through collaborative research involving HCPs, researchers, and the supplier company, rather than being presented as a finalized product. While the collaboration with supplier companies was deemed valuable and appreciated, it was also described as complex due to a lack of a common language and challenges in understanding each other’s perspectives.

While apps that could be easily downloaded to patients’ own private devices were seen as convenient, health care–provided encrypted devices such as those in the eCH approach were viewed as offering easily accessible communication without security concerns. The HCPs stressed the importance of user-friendly interventions to prevent the use of the family’s private technology, such as FaceTime (Apple Inc./AT&T Inc.) on smartphones for video meetings or texting. Furthermore, a larger-sized tablet, such as the one used in eCH, was preferred for video calls over a smartphone. However, using a tablet for photography or having to carry an additional device was seen as less convenient for the patients.

The HCPs highlighted the necessity for ongoing technological development to seamlessly integrate eHealth tools such as eCH with existing electronic systems, particularly medical records. This integration would help reduce the burden of duplicated work or data transfer while ensuring compliance with data protection and patient data laws [21].

One feels that one might be less careful with security aspects if I am to start transferring photographs to my computer and then print them and then scan them to somewhere.

Interview 2 staff

The HCPs believed that many of the challenges related to intersystem information and data transfer would be resolved with the implementation of a new regional system (ie, the Scania [a region in Sweden] digital health care system or SDV). This system would offer unified patient records and facilitate the connection of mobile devices, medical equipment, and imaging to support home care.

### Domain 3: The Value Proposition

The HCPs highlighted that eCH provided various communication channels that could be tailored to individual preferences. This approach was seen as empowering families and allowing them to feel more engaged and in control of their care. eCH facilitated 2-way, asynchronous communication, enabling families and staff to interact without the risk of inconveniencing each other during potentially busy periods, as telephone calls might do. eCH was viewed as an efficient and convenient means of communication.

The HCPs also emphasized the importance of eHealth communication, with eCH being one example, in terms of promoting equitable and inclusive health care. They pointed out that while telephone calls might exclude or bypass children, text or video chats allowed for more direct communication with the child and a better understanding of their situation. Chat messages were seen as a way to facilitate small talk and build rapport. Additionally, follow-up care for children with chronic or long-term illnesses could be conducted through quick text messages, reducing the need for frequent hospital visits. The HCPs believed that this approach enabled children to communicate with health care providers confidentially, on their own terms, and without parental involvement, potentially giving them more responsibility and a sense of ownership over their care.

We were a bit surprised/overwhelmed by the positive response we got from the children. It was like: This is my app. I want my own. You cannot write in my app. I’m the one who is writing there [not you].

Interview 4 staff

The HCPs also discussed how eHealth could empower children to become more involved in their prehospital care. For instance, they mentioned the possibility of children filling out electronic questionnaires at home or participating in virtual tours of health care facilities and procedures to help them feel more prepared for their hospital visits. This approach was seen as leading to better care outcomes, with children feeling more confident and less anxious. Over the long term, the HCPs believed that eHealth could be used to gradually provide children with chronic illnesses with more information and responsibility, better preparing them for the eventual transition to adult care.

The HCPs also highlighted how eHealth streamlined their work by offering a comprehensive view of patients and their care needs. They particularly valued electronic reports, which provided data on patients’ weight, health status, and pain through eHealth. Additionally, the use of eHealth for communication was seen as a way to bridge the gap between research units and health care facilities across Sweden. HCPs at national health care centers could participate in digital 3-party meetings with families and other HCPs at local hospitals, fostering collaboration and knowledge-sharing.

When there was a patient who was going to meet the doctor at the local hospital we participated via the tablet. And I hope to be able to do that more in the future...They were going to support us with some of the child’s care. So, we came along, and we could exchange experiences and we could support a bit. So that was nice. To be able to feel as one [unit] all over the country.

Interview 2 staff

The HCPs emphasized that eHealth should not be viewed as a panacea for health care challenges but rather as a supplementary tool that offers flexible and effective communication. They discussed the complexity of maintaining patient privacy and the confidentiality of children in particular. Providing digital care for young children and infants was seen as less complex, but when dealing with adolescents, the same interventions could both challenge and strengthen the care relationship. Safeguarding the child’s privacy was a concern, especially during video conversations through a tablet or computer at home, where the child might not want to share everything with their parents, and they should have the right to withhold information. The HCPs also expressed concerns about the reduced ability to identify cases of unhealthy domestic environments in digital interactions, as the nuances of verbal and nonverbal communication were lost.

### Domain 4: The Adopter System

The HCPs noted that children and parents are generally proficient in adopting and using digital technologies, sometimes even more so than health care staff. However, they emphasized the need to align the expectations of families regarding eHealth with individual circumstances. For example, reporting data through eCH required patients and family members to take on tasks typically performed by HCPs. The HCPs stressed the importance of eHealth aligning with valid and reasonable expectations from the child and their family while minimizing the effort required from families. They emphasized that family needs should determine the extent of eHealth use, with careful consideration of when and what to offer digitally versus in person. Establishing a relationship digitally was seen as challenging, and the HCPs recommended that the nursing relationship be initially established in person and then continued digitally when beneficial.

The HCPs discussed the necessity of adapting their work practices to incorporate eHealth into their regular schedules. This adaptation involved more frequent contact with each patient and an increase in working hours dedicated to digital communication. HCPs also mentioned that they had established scheduled daily time slots for eHealth communication to minimize interruptions and gain better control over their workday. They emphasized the need to strike a balance between additional tasks related to both physical and digital care to ensure manageable workloads and maintain the quality of care. Managing conversations with multiple families concurrently required a strategy for shifting attention and a logistical system that did not lead to stress.

It implies a different way [to work] that we might not have found to hundred percent yet,.... Because one would typically often start the morning by checking the eCH. And at that time, it is not a problem. But then one wants to check at least two more times, to be able to respond somewhat regularly and keep up the dialogue. I think I have had as many as nine tablets operating in parallel. And then, this were like, some days, as if I would have nine extra physically present which was a bit...It takes some juggling.

Interview 2 staff

The HCPs explained that various eHealth devices served distinct purposes, either sequentially or concurrently. For instance, eCH was used for 2-way communication, while 1177 [22], a national health care hub in Sweden providing advice, information, and health services via phone and web, was primarily used for administrative functions such as appointment cancellations, transferring certificates for parental allowances, and prescribing medications. The HCPs emphasized the importance of being mindful of the potential for an overload of apps and the need to evaluate whether a new eHealth initiative was necessary or if existing digital communication systems could fulfill the same function.

It is a balancing act...and then the teenaged patient would have said something like I cannot take any more apps. I do not want another app. I´m tired of apps! That’s also a view to take with you. Is there a limit? What apps should be prioritized and what should not be prioritized app-wise so to speak, and in digital form?

Interview staff 4

### Domain 5: The Organization

In general, the HCPs expressed optimism regarding the organization’s preparedness and ability to transition toward enhanced digital care. They noted that the COVID-19 pandemic had accelerated this readiness. They also had confidence in the future funding of eHealth initiatives. Furthermore, being part of a research program supported the initial stages of implementation by providing additional time or necessary human resources. The HCPs identified high user value and a clearly defined purpose as the primary facilitators for implementing eHealth within the organization, particularly when extra effort or patience was needed.

The HCPs highlighted various factors influencing the organization’s readiness to implement eHealth. These factors included individual attitudes, preconceived notions, and the age of the HCPs involved in the implementation. They emphasized that the shift toward increased eHealth usage required time and effort, as sticking to familiar routines might seem more comfortable and less costly in the short term. They mentioned that they often settled for using only a few features, such as utilizing the chat function in eCH, even though video conversations were recognized as valuable and readily available. The HCPs believed that a positive collective experience with eHealth, such as their involvement in the research project (eCH), encouraged readiness and reduced resistance to future innovations.

The HCPs also pointed out the absence of a unified strategy and alignment within the hospital concerning eHealth. They noted that various parts of the organization were at different stages of readiness for change. A fragmented organizational structure with separate management for nursing and medical staff was identified as an obstacle to establishing partnerships, leading to a slow-moving system that operated in isolation. Therefore, they emphasized the need to enhance coordination and collaboration to fully leverage existing eHealth initiatives and prevent redundant efforts.

They highlighted the necessity for a revised care model to facilitate the implementation of eHealth. They discussed the need to broaden the definition of health to encompass meaningful communication between parties, considering it as a service provided from 1 party to another in various ways and forms. They emphasized the necessity of redefining and renegotiating communication norms to integrate eHealth initiatives as a seamless component of regular professional health care services. They mentioned that if eHealth was perceived as an ancillary activity, it might be at risk of being deprioritized in comparison to physical tasks or regarded as an additional responsibility for nurses without a clear purpose. The health care providers described an ongoing challenge in achieving a point where digital meetings could be formally recorded as regular visits and recognized as a documented activity. They emphasized the importance of management not expecting to save time by implementing eHealth but rather to enhance the quality of care. They emphasized the importance of organizational-level reflection regarding how changes, such as reducing physical visits, would impact the necessity for other modifications in care delivery and how this might influence the need for facility adaptations.

There can be six people in that room at the same time as one is active with communicating on the tablet.

Interview 3 staff

They were concerned that if eHealth were implemented without careful planning, it could result in increased workloads and heightened expectations from families for greater access to care, for which the organization was unprepared.

Research projects with stringent inclusion criteria focused on a minority of patients, such as children with specific diagnoses or ages, and those that only included certain aspects of care through eHealth, were seen as more challenging to integrate into routine care. The HCPs shared experiences of how implementation became easier when someone was willing to take the lead in the everyday clinical implementation, which others could then follow. Having enthusiastic individuals in place was described as a key factor for successful implementation and for providing technical support. By contrast, unclear responsibility or purpose of care could make the implementation of research in clinical care more complex. According to HCPs, a perceived lack of support functions for IT systems could make implementation difficult and reduce the capacity for change. Thus, the need for time to discuss challenges and errors was emphasized as important for facilitating implementation.

### Domain 6: The Wider Context

The HCPs mentioned the strict regulatory requirements for health care documentation, emphasizing the need for careful consideration of how to handle information produced through the usage of new eHealth interventions. A significant part of the material produced through eCH, such as text chat messages, was unsuitable or not intended for patient records. However, they emphasized the importance of actively considering the regulatory framework when deciding how to handle produced material, such as text, photographs, and reported health care data. When the pandemic started in Sweden, eCH was described as extremely useful in departments where the project was already well integrated into care (pediatric surgery and neonatology). However, other departments were at earlier stages in the process, with ongoing development or just beginning patient inclusion (oncology and cardiology). For these departments, the pandemic became a hurdle, limiting time for project meetings and leading to changed prioritizations within the organizations, ultimately putting the research on hold.

Public procurement of eHealth services originating from a research project was described as a time-consuming and challenging process, involving significant effort in terms of preparation and communication. The HCPs expressed frustration with an unclear and slow procurement process, even though there was a high sense of urgency to ensure the continued delivery of eHealth services such as eCH, which had become vital for the organization. They were concerned about the risk of service interruption during the transition from research to clinical practice.

There are rules regarding public procurement. And then there is technology, and you have to turn to the IT-department. And then you get all sweaty.

Interview 1 management

The ambition of Sweden to become a leader in eHealth was considered in light of the evolving nature of digital communication as the norm, especially among the younger population. However, this had to be balanced with the strict requirements for safety and confidentiality in health care. The HCPs highlighted the challenges related to data safety, particularly in collaborations with suppliers, where there were uncertainties about what was allowed and feasible. For instance, certain age restrictions on digital IDs for children were seen as a complicating factor in the use of eHealth.

### Domain 7: Embedding and Adaptation Over Time

The HCPs explained that eHealth was in a constant state of development, offering numerous possibilities for changing and enhancing health care. They observed that different systems evolved into more complex and versatile tools, each having its unique challenges, errors, and strengths. Some eHealth systems seemed to seamlessly integrate into daily routines, while others disappeared discreetly. In some cases, the HCPs mentioned that they adopted these systems without a deep understanding of why or how it was done, although they emphasized the importance of management support and dedicated time as critical factors in this process.

It takes managers who believe in it and are willing to devote time and provide opportunity. To have the energy to think long-term. That’s very important because there are always running-in problems and inexperience. And it is easy to give up in the beginning no matter what it is. But if there is an understanding to set aside time to learn. Then it will become an established way of working.

Interview 5 management

They highlighted the importance of involving a critical mass of staff in the implementation of an eHealth intervention to reduce the risk of unforeseen errors. Additionally, they stressed that considerations regarding eHealth should be integrated into the early planning stages of future care to allow for necessary reflections, which could be crucial for successful adaptation.

The HCPs identified a lack of organizational oversight regarding available eHealth services and limited efforts to share experiences and build upon them as significant barriers to long-term adoption.

## Discussion

### Principal Findings

We used the NASSS framework [8] to analyze conditions for the implementation of an eHealth intervention, eCH, in pediatric care. The starting point of our study was a local, bottom-up development project initiated by HCPs in collaboration with researchers. Our findings indicated a mix of simple, complicated, and complex conditions for the implementation of eHealth, which was generally perceived to have a high value within the context of pediatric hospital care. Barriers to implementation tended to be on a rather detailed level, regarding what is safe and allowed, given the heavy regulatory requirements in health care. In addition, results indicated the need to establish a shared vision for eHealth-related work to become part of regular health care. This points toward the need for bottom-up eHealth initiatives to connect to a wider group of stakeholders within their institutional setting. These results also point toward a need for organizational eHealth strategies compatible with legal, technological, and security concerns. First, we discuss circumstances that contribute to relatively simple conditions favorable to adoption and scaling up according to the NASSS framework. Second, circumstances contributing to increased complexity are discussed.

### Facilitation of Adoption and Scaling Up

Aspects such as ease of use, adaptability, small scalability, and specificity with a high perceived value regarding both the services and the patients contributed to the classification of eCH as simple with significant potential for adoption and scaling up. eHealth interventions such as eCH have a great potential to complement other forms of communication through increased flexibility, access to care, and a perceived sense of security for families caring for children at home. These notions are also recognized from previous research based on interviews with parents evaluating their use of eCH [16,18]. Hence, the perceived value of eHealth appears to be relatively coherent for patients and HCPs.

Front-line staff encountered the need for changes in routines and work organization early on, requiring support from management to address these challenges. A key factor in dealing with these processes is the active contribution of enthusiasts and project leaders who can identify obstacles and bring stakeholders together to facilitate the process. The extent to which organizational units have had the opportunity to participate in the development of digital tools dedicated to well-defined groups of patients and tailored to the demands of both patients and HCPs, such as eCH [16], appears crucial for continuation and adoption.

The pandemic appeared to have sped up the innovation and change in the services at hospital departments where eHealth was already up and running. Nevertheless, the implementation of eHealth during a pandemic presented a substantial challenge for departments that were not fully prepared before the outbreak, resulting in the need to temporarily halt project-related work. Previous research on the adoption of eHealth services across Europe during the coronavirus pandemic highlights various factors that could diminish the public value of eHealth [23]. Therefore, proceeding cautiously in the face of rapidly changing circumstances may be crucial to prevent compromising quality and the impact on public health.

### Potential Barriers to Adoption and Scaling Up

HCPs expressed a preference for digital interventions that streamlined their workflow and could be seamlessly integrated with other systems such as medical records. However, they noted that this integration was a complex challenge that required careful navigation. The complex and sometimes conflicting demands of complying with health care regulations concerning documentation, patient safety, and information security, combined with aging systems not aligned with ongoing development projects, can undermine the realization of eHealth initiatives. While many of the strengths and positive experiences of new digital tools and eHealth were related to participation in development and implementation, there was less confidence in the hospital and region’s capability to provide a context for learning and adaptation over time. Indeed, there are very few examples of familiarity with strategies, guidelines, or interfaces between ongoing bottom-up activities (such as eCH) and the general architecture of eHealth at the hospital and in the region [24]. This observation is in line with previous research that showed that HCPs can be motivated to pursue development despite a lack of alignment in this regard [13].

The handling of patient-generated health care data collected in everyday environments from patients would require new standards as well as technical and organizational support to ensure that the data are well-managed and tailored toward clinical objectives to ensure success [25]. The relatively loose coupling and provisional supplier model in the research project provided flexibility and was advantageous for the project, but it introduced uncertainties in the long run. Challenges related to procurement and delays in large IT projects within the regional health care organization appeared to create an atmosphere of uncertainty and, in some cases, cynicism among health care providers. Effective public procurement of eHealth requires specialized skills regarding, for example, interoperability and life-cycle costing [26].

The HCPs described the pediatric health care population as heterogenous in age and maturity with a capacity for autonomy that changes over time, implying a need for the continuous adaptation of routines. Sociotechnical aspects related to potential changes in routines, work division, and workload were often postponed to a later phase of implementation. Over time, this somewhat fragmented approach may elevate the complexity of implementation and place excessive demands on both patients and health care providers. Besides, eCH may be based on strict inclusion criteria and may exclude patients in terms of sociocultural factors. To ensure equity and participation, the implementation of eHealth should address cultural hurdles [27]; for example, in the case of eCH, this can be achieved by incorporating adaptations such as interpreter services.

It is vital that the integration of eHealth includes considerations related to secure communication and privacy in traffic and data transmission [28]. It can be argued that this difference constitutes a sort of “app-gap” leading to increased risk for improvisation, cutting corners through using private technology rather than complying with the digital tools provided by the hospital. Such app-gaps may also contribute to frustration, negative attitudes, and diminishing legitimacy for IT governance and information security.

Our findings support previous research in that the organizational capability for adoption and scaling up is dependent on sophisticated collaboration and alignment between professions, clinics, and divisions within the hospital, which contributes to complexity and vulnerability [8,29]. This needs to proceed from a shared view and definition of health care that incorporates eHealth-related work as health care provision. Organizations that depend on resources provided by research and development projects to engage in new eHealth technologies risk adding extra workload to staff. In this perspective, it is important to be able to identify the true value of eHealth beyond economic terms [30].

### Strengths and Limitations

This study incorporates both staff and managers’ experiences and views of eHealth. Although the sample size was small, it comprised staff with deep familiarity and insight into both eCH and their respective specialties as well as different management levels in pediatric care. Experiences shared related to both the specific project and eHealth in general. This was deemed important and a strength of the project because development, evaluation, and implementation take place in an organizational context in which different eHealth interventions and innovations are ongoing, coexisting, and coevolving over time. The study was performed during the COVID-19 pandemic, which increased the study period and likely reduced the number of participants. Furthermore, the study was conducted in 1 of 21 regions in a small, high-income country with a nationally regulated and regionally provided health care system. While the country, in general, has a high level of digitalization, health care continues to struggle with the standardization and integration of eHealth. These perspectives may be considered in relation to the transferability of the results. The results fit well into the domains of the NASSS framework for implementation and contribute to a holistic and contextual perspective, which may support the translation of eHealth research into policy and practice [31].

### Conclusions

The study enhances our comprehension of how eHealth initiatives can harmonize with the organizational environment within their specific context, thereby enhancing the potential for adoption and scalability. It further adds to the body of research indicating that the NASSS framework has the potential to be of great use in the planning and coordination of eHealth development in health care settings [32,33]. While studies on implementation are mainly concerned with difficulties in reaching out, changing, and institutionalizing new behaviors, this study indicates the need for a much broader approach. Thus, there is a need to establish networks and communication channels between staff, managers, IT professionals, legal departments, researchers, and supplier companies [25]. This study should be complemented with research into how specific eHealth interventions are perceived by the market, regional information and communications department, procurement, and information security professionals at the hospital and in the region. The possible business case or value for suppliers or investors in eHealth was not addressed by participants in this study, although it had a significant impact on implementation according to NASSS [8]. Deepened knowledge about HCPs’ understanding of, and collaboration with, a wider eHealth innovation system could thus further support sustainable implementation.

## Abbreviations

eCH: eChildHealth
HCP: health care professional
NASSS: Non-adoption, Abandonment, and Challenges to the Scale-Up, Spread, and Sustainability of Health and Care Technologies
